# Electronic and Optoelectronic Applications Based on 2D Novel Anisotropic Transition Metal Dichalcogenides

**DOI:** 10.1002/advs.201700231

**Published:** 2017-10-06

**Authors:** Chuanhui Gong, Yuxi Zhang, Wei Chen, Junwei Chu, Tianyu Lei, Junru Pu, Liping Dai, Chunyang Wu, Yuhua Cheng, Tianyou Zhai, Liang Li, Jie Xiong

**Affiliations:** ^1^ State Key Laboratory of Electronic Thin Films and Integrated Devices University of Electronic Science and Technology of China Chengdu 610054 P. R. China; ^2^ School of Automation Engineering University of Electronic Science and Technology of China Chengdu 610054 P. R. China; ^3^ State Key Laboratory of Material Processing and Die & Mould Technology School of Materials Science and Engineering Huazhong University of Science and Technology Wuhan 430074 P. R. China; ^4^ College of Physics, Optoelectronics and Energy Center for Energy Conversion Materials & Physics (CECMP) Soochow University Suzhou 215006 P. R. China

**Keywords:** 2D, anisotropy, electronics, optoelectronics, transition metal dichalcogenides

## Abstract

With the continuous exploration of 2D transition metal dichalcogenides (TMDs), novel high‐performance devices based on the remarkable electronic and optoelectronic natures of 2D TMDs are increasingly emerging. As fresh blood of 2D TMD family, anisotropic MTe_2_ and ReX_2_ (M = Mo, W, and X = S, Se) have drawn increasing attention owing to their low‐symmetry structures and charming properties of mechanics, electronics, and optoelectronics, which are suitable for the applications of field‐effect transistors (FETs), photodetectors, thermoelectric and piezoelectric applications, especially catering to anisotropic devices. Herein, a comprehensive review is introduced, concentrating on their recent progresses and various applications in recent years. First, the crystalline structure and the origin of the strong anisotropy characterized by various techniques are discussed. Specifically, the preparation of these 2D materials is presented and various growth methods are summarized. Then, high‐performance applications of these anisotropic TMDs, including FETs, photodetectors, and thermoelectric and piezoelectric applications are discussed. Finally, the conclusion and outlook of these applications are proposed.

## 1 Introduction

Since the discovery of graphene decades ago, 2D materials have been studied by many research groups because of their excellent mechanical, electronic, and optoelectronic properties.^1^, ^2^, ^3^, ^4^ 2D transition metal dichalcogenides (TMDs) show great promise for the following reasons. First, their monolayers are bonded via van der Waals (vdW) interactions. In the construction of heterostructures based on TMDs, mismatches between the thermal and lattice coefficients of different materials can be avoided.^5^, ^6^ Second, the high mobility and 2D nanostructures of TMDs endow them with great electronic performances, including enhanced integration levels and suppressed short‐channel effects.^2^, ^7^, ^8^, ^9^, ^10^ Third, the indirect–direct bandgap transition between the bulk and monolayer in some TMDs affords various optoelectronic applications, from photodetectors to light emitters.^5^, ^11^, ^12^, ^13^ The common chemical formula of TMDs is MX_2_, where M is a transition metal (group IVB–VIIB; M = Mo, W, Re, and so on) and X is a chalcogen (group VIA; X = S, Se, Te).^14^, ^15^ The sandwich structure of TMDs leads to excellent electronic and optoelectronic properties.^14^, ^15^, ^16^, ^17^, ^18^ Currently, TMDs exhibit satisfying properties in various applications.^19^, ^20^, ^21^, ^22^ Ultralow standby power dissipation was realized in single‐layer MoS_2_‐based field‐effect transistors (FETs), with a high on/off ratio of 10^8^.^2^ Furthermore, ultrasensitive photodetectors based on monolayer MoS_2_ were demonstrated by Lopez‐Sanchez et al.^23^ And high efficiency light‐emitting diodes (LEDs) based on monolayer WSe_2_ p–n junctions via electrostatic doping of gate were investigated by Baugher et al.,^24^ Ross et al.,^25^ and Pospischil et al.,^26^ respectively. In addition, the transparent and flexible nature of TMDs makes them ideal candidates for flexible electronic devices.^9^, ^15^, ^17^ However, the studies of current 2D materials such as black phosphorus, group IV monochalcogenides (SnS, SnSe, GeSe, GeS), and anisotropic TMDs (WTe_2_, ReX_2_) are starting to focus on their anisotropic properties.^1^ It is beneficial to control variability and uniformity of device performance by manipulating crystal orientation.^1^, ^27^ In addition, this novel physical degree of freedom can be applied for polarized light detection devices and potential valleytronics.^28^, ^29^, ^30^ Herein, we discuss the recently developed MTe_2_ and ReX_2_ materials, which exhibit anisotropic features originating from their unique in‐plane atomic arrangement.

The strong anisotropic properties of the MTe_2_ and ReX_2_ materials discussed in this paper originate from their distorted octahedral phase, specifically the distorted 1T phase for MoTe_2_ and WTe_2_, and the strong metal–metal bond (Re—Re bond) in the 1T′ phase for ReS_2_ and ReSe_2_. Researches based on Raman spectroscopy, polarization‐resolved reflectance spectroscopy, and photoluminescence spectroscopy have demonstrated the strong anisotropic characteristics of these materials.^31^, ^32^, ^33^ In addition to their anisotropic properties, these materials have many other fascinating properties. The ultrahigh theoretical carrier mobility (1000–2000 cm^2^ V^−1^ s^−1^) of MTe_2_ and ReX_2_ facilitates the ultrahigh‐speed conversion of photons into electrical signals, providing an ultrafast response time in optoelectronic devices.^8^ Furthermore, monolayer MTe_2_ and few‐layer ReX_2_ possess direct energy gaps from the near‐infrared to the visible spectral region, as their bandgap ranging from 1–1.6 eV.^1^, ^34^, ^35^, ^36^, ^37^, ^38^, ^39^, ^40^ Unlike other symmetrical 2D materials, such as graphene and MoS_2_, MTe_2_ and ReX_2_ have asymmetrical 2D crystal lattices. In‐plane orientation‐dependent electron and phonon properties are introduced into the system, which may inspire new concepts or ideas in angle‐resolved semiconductor devices.^1^, ^41^, ^42^, ^43^ Zhang et al. predicted that the valley degree of freedom, a key factor in valleytronics, can be tuned in MoTe_2_ due to its anisotropic structure, as determined by first‐principle calculations.^29^ In addition, a new linear dichroic photodetector based on ReS_2_ with a high responsivity of 1000 A W^−1^ was reported by Liu et al.^44^

In this review article, we give a comprehensive review of the recent progress in anisotropic MTe_2_ and ReX_2_, with particular focus on their various applications. First, the crystalline structure and the origin of the strong anisotropy of these materials were analyzed using various characterization techniques. In addition, we discuss the preparation of these 2D materials, highlighting relevant mechanical exfoliation and chemical vapor deposition (CVD) methods. Following this, we discuss FETs, photodetectors, thermoelectric and piezoelectric applications based on these anisotropic TMDs. Finally, we present the challenges, opportunities, and outlook of these applications based on current views.

## 2 Anisotropic Crystalline Structure

### 2.1 Crystalline Structure

**Figure 1:**
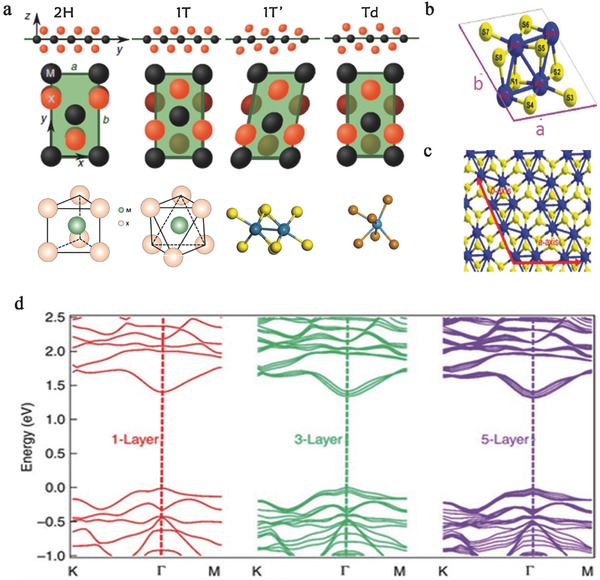
a) The four crystalline phases (2H, 1T, 1T′, Td) of 2D TMDs. M represents a metal atom, X represents a chalcogen compound, and all three phases are composed of the X—M—X layer structure. Reproduced with permission.^33^ Copyright 2014, Nature Publishing Group. Reproduced with permission.^45^ Copyright 2015, American Chemical Society. Reproduced with permission.^65^ Copyright 2015, American Physical Society. b) Perspective drawing of a primitive cell of monolayer ReS_2_. The blue shows the strong metallic Re—Re bond. Reproduced with permission.^66^ Copyright 2015, American Physical Society. c) crystal structure of monolayer ReS_2_, the red arrows represent the direction of *a* and *b* axes. d) Band structure of monolayer, trilayer, and five‐layer ReS_2_. Reproduced with permission.^67^ Copyright 2015, the authors, published under CC‐BY‐4.0 license.

TMDs possess a layered crystal structure with individual layers bonded together via weak van der Waals interactions.^37^, ^45^, ^46^, ^47^, ^48^, ^49^, ^50^ Typically, for TMDs, six chalcogenide (X) ligands are covalently bonded to one transition metal atom (M) center such that no dangling bonds appear in each monolayer. Due to their atomically flat surface, TMDs are ideal materials for application in novel FETs in which the short‐channel effects are optimized.^49^, ^51^, ^52^, ^53^, ^54^ Under ambient conditions, most well‐studied group VI TMDs, such as MoS_2_, have an isotropic hexagonal phase (2H) and octahedral phase (1T) crystal structure. This structure consists of alternating stacks of single‐layer trigonal prisms formed by X atoms around the M atoms (**Figure** **1**a). In general, the physical properties of TMDs are closely related to their lattice structures and the in‐plane configuration of different atoms.

The lattice structures of some TMDs that have recently joined this family, specifically MTe_2_ and ReX_2_, can be quite different. Unlike MoS_2_, these four materials exhibit a distorted structure relative to the 1T phase. The monolayer of the distorted 1T phase still has an X—M—X structure, where the upper X atoms are rotated by 180° with respect to the lower X atoms, forming an M‐centered octahedral.^33^, ^55^, ^56^ The center M atoms experience a shift in the layer plane along the perpendicular direction, forming strong metallically bonded X pairs. Additionally, the X atoms are no longer coplanar, but instead exhibit a zigzag structure varying along the atomic positions of the perpendicular direction. Small variations in the displacement and stacking lead to two different phases: the 1T′ phase (ReS_2_, ReSe_2_, and MoTe_2_ in their natural state) and the Td phase (WTe_2_ in its natural state).^40^, ^46^, ^48^, ^54^, ^57^, ^58^, ^59^, ^60^, ^61^, ^62^ We show the 2H, 1T, 1T′, Td crystal structures in Figure 1a. And the parameters of typical TMDs were shown in **Table** **1**. This symmetry‐reducing effect strongly enhances the anisotropy in the unit cell. Thus, compared with the well‐studied TMD materials, these four 2D materials show astonishing characteristics in optoelectronic and electronic applications.

**Table 1:** Parameters of typical TMDs^8^, ^16^, ^189^ (ML: Monolayer, E: Experimental value, C: Calculated value)

TMDs	Crystal structures	Types	Bandgaps (Bulk/ML) [eV]	Carrier mobility [cm^2^ V^−1^ s^−1^]	References
MoS_2_	1T/2H	n‐type	1.2 (ind.)/1.8 (dir.)	15–60 (E) 340 (C)	2, 69, 70, 71
MoSe_2_	1T/2H	p‐type	1.4 (ind.)/1.58 (dir.)	≈50 (E) 240 (C)	73, 74
MoTe_2_	1T/1T′/2H/Td	p‐type	0.88 (ind.)/1.10 (dir.)	25–68 (E) 2526 (C)	34, 35, 36, 75, 76
WS_2_	1T/2H	n‐type	1.4 (ind.)/2.1 (dir.)	50–180 (E) 1103 (C)	77, 78, 79
WSe_2_	1T/2H	p‐type	1.2 (ind.)/1.65 (dir.)	250 (E) 705 (C)	80, 81
WTe_2_	1T/1T′/Td	Semimetal	–	–	64, 82
ReS_2_	1T′/Td	n‐type	1.5 (dir.)/1.58 (dir.)	30–40 (E)	24, 40, 83, 84, 85, 86
ReSe_2_	1T′	p‐type	1.27 (dir.)/1.24 (ind.)	4–11 (E)	48, 53, 87, 88, 89

Moreover, the crystalline structures in these strongly anisotropic TMDs are not static. For MTe_2_ and ReX_2_, the 1T′ or Td phase of the atomic system is stable for the ambient environment. However, under certain conditions, the 1T′ (or Td) and 1T phases interconvert, as the energy gap of the two phases can be bridged mechanically or thermally.^63^, ^64^ However, though the rhenium atom contains an extra electron in the d orbital, there are still some differences between polytelluride TMDs (MoTe_2_/WTe_2_) and group VI TMDs with rhenium atoms (ReSe_2_/ReS_2_) in the crystalline structure, which is discussed in detail in the following section.

#### 2.1.1 Anisotropy of Group VI TMDs with Rhenium Atoms

In contrast to traditional TMDs, such as MoS_2_, the chalcogen atoms are not all equally displaced above and below the Re plane in polytelluride TMDs.^90^, ^91^, ^92^, ^93^, ^94^, ^95^ In this special 1T′ crystalline structure, the extra electron in the d orbital of the rhenium atom promotes the construction of a strong metallic Re—Re bond (Figure 1b).^32^, ^38^, ^66^, ^96^ The distance between these dimerized Re atoms can be even shorter than that in rhenium single crystals^97^; thus, the total energy and symmetry of the system are reduced.^40^ This is a major origin of the strong anisotropic properties of ReSe_2_ and ReS_2_ validated by Liu et al.^67^ The in‐plane anisotropic properties of monolayer and few‐layered ReS_2_ are shown in Figure 1c, ReS_2_ has two principal axes, (*a* axis, 118.97°; *b* axis, 61.03°). In accordance with the angles of the *a* and *b* axes, a quadrilateral shape with an angle of ≈60° or 120° is observed in thin, exfoliated ReS_2_, and the large anisotropic ratio of mobility of ReS_2_ along the two axes (up to 3.1) is the highest of all studied layered 2D materials. Meanwhile, the weak interlayer interaction exhibited in ReS_2_ and ReSe_2_, which is different from other TMDs, also affects the strong anisotropic properties.^65^ As determined by measuring the layer thickness, the ReS_2_ crystal possesses an interlayer coupling weaker than most TMD crystals, such as MoS_2_ and WS_2_. The interlayer decoupling originates from the Peierls distortion of the initial 1T ReS_2_ revealed by Zhong et al. As a result of the disordered stacking and minimized wavefunction overlap, this interlayer decoupling provides an ideal platform to characterize unique phenomena in few‐layered ReX_2_ while circumventing the challenge of preparing monolayer samples.^65^ Tongay et al. also confirmed that because of the weak interlayer interactions between the distorted Re atoms, the van der Waals forces and the binding energy between the ReS_2_ layers are very weak.^40^ Therefore, both bulk crystal and monolayer ReS_2_ exhibit unique anisotropic optical and electronic properties.^40^, ^97^ In particular, monolayer ReSe_2_ (or ReS_2_) has fascinating anisotropic properties at near‐infrared frequencies. Yu et al. calculated that ReS_2_ maintains a direct bandgap (≈1.58 eV) from bulk to monolayer with no dependence on the number of layers based on density functional theory (DFT) (Figure 1d).^38^, ^40^, ^74^ And the monolayer possesses strong in‐plane anisotropy with vastly different quantum confinement effects and a rich Raman spectrum, demonstrating its great potential as an optoelectronic material. For ReSe_2_, the monolayer is observed to be an indirect bandgap semiconductor at the DFT level. Nevertheless, electron–electron interactions in ReSe_2_ lower the valence band energy and shift the valence band maximum. This makes monolayer ReSe_2_ a direct bandgap semiconductor.^65^

#### 2.1.2 Anisotropy of Polytelluride TMDs

**Figure 2:**
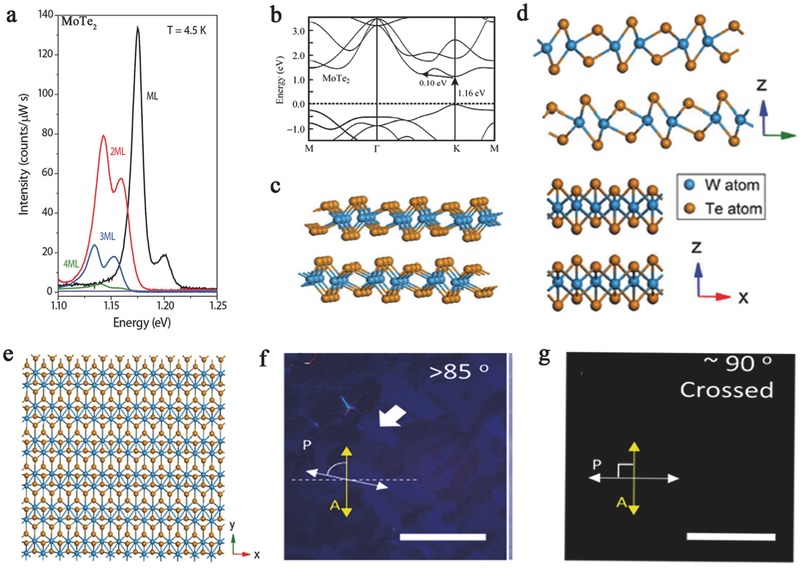
a) The bandgap structure of MoTe_2_ from four layers to monolayer. Reproduced with permission.^35^ Copyright 2015, American Chemical Society. b) The bandgap of monolayer MoTe_2_ is 1.16 eV. Crystalline structure of Td WTe_2_. Reproduced with permission.^8^ Copyright 2014, Springer. c) Perspective view. d) Front view and side views. e) Top view. The grains and grain boundaries of monolayer MoTe_2_ were observed by polarized optical microscopy. Reproduced with permission.^99^ Copyright 2016, the authors, published under CC‐BY‐4.0 license. f) The grain can be clearly observed in 5°–10° of the polarizer rotation near 85°. g) Under perfectly crossed polarizer–analyzer (P–A) setup, nothing can be seen. Reproduced with permission.^98^ Copyright 2014, IOP Publishing.

For 2D tellurides, specifically MoTe_2_ and WTe_2_, the development of anisotropy is complicated due to their rich phase diagrams. MoTe_2_ (WTe_2_) possesses semiconducting (2H) and semimetal distorted octahedron (1T′ or Td) phases and exhibits anisotropic absorption behavior in the visible range.^98^ For MoTe_2_ and WTe_2_, the strong anisotropic properties lead to a semimetal phase. The stable Td phase of WTe_2_ imparts reduced symmetry, which leads to the in‐plane anisotropy of various physical properties.^99^ MoTe_2_ exhibits an indirect–direct bandgap transition when going from bulk to monolayer (**Figure** **2**a,b).^99^, ^100^, ^101^ For monolayer MoTe_2_ flakes, the alternating Te atoms along the dimerized Mo atoms of 1T′ MoTe_2_ 102 generates different torsion angles depending on the scan direction. Keum et al. predicted in their previous work that the typical monoclinic bulk 1T′ MoTe_2_ phase is less stable than the 2H phase. However, by dimerizing a series of Mo atoms along the (10) direction, Keum et al. achieved a stable monoclinic 1T′ MoTe_2_ phase; this process is known as the 1D Peierls distortion of MoTe_2_.^103^ Tungsten ditelluride (WTe_2_) is a semimetallic layered transition metal dichalcogenide in the Td phase. Td WTe_2_ is constituted of triple‐layer covalently bonded Te—W—Te atomic planes stacked along the perpendicular direction, as shown in Figure 2c,d. Td WTe_2_ is strongly distorted from the ideal hexagonal net because the off‐centering W atoms form slightly buckled W—W zigzag chains along the *x* axis of the orthorhombic unit cell (Figure 2e). Han et al. investigated the anisotropy of 1T′ MoTe_2_ and Td WTe_2_ through absorption spectroscopy and polarizing optical microscopy. 1T′ MoTe_2_ (or Td WTe_2_) showed strong anisotropic properties, and exhibits anisotropic absorption behavior in the visible range. Figure 2f shows the polarizing optical image with enhanced contrast by inserting both a polarizer and analyzer crossed by nearly 85°.^98^ This phenomenon can be ascribed to anisotropic absorption. In addition, when the crossing angle is 90°, the light transmission disappears (Figure 2g).

### 2.2 Raman Spectra of Strongly Anisotropic TMDs

**Figure 3:**
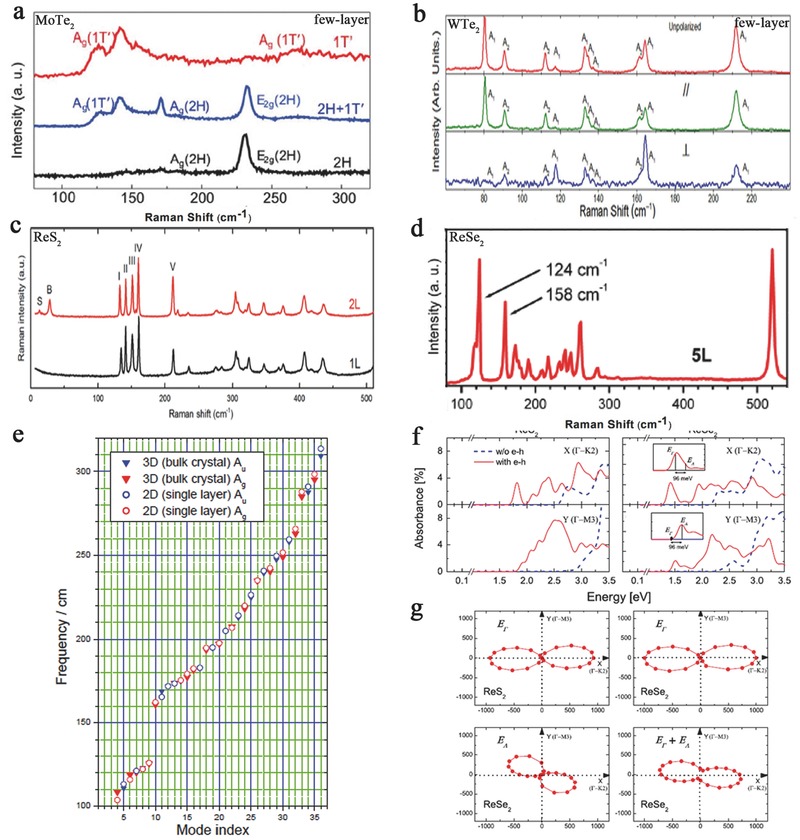
a) The Raman spectra of the 2H phase (black) and the 1T′ phase (red) of MoTe_2_. The Raman spectra of from mono‐ to few‐layers of the TMDs in the 1T′ (or Td) phase. Reproduced with permission.^105^ Copyright 2015, American Association for the Advancement of Science. b) WTe_2_. Reproduced with permission.^99^ Copyright 2016, the authors, published under CC‐BY‐4.0 license. c) ReS_2._ Reproduced with permission.^108^ Copyright 2016, American Chemical Society. d) ReSe_2_. e) Scatter plot of the vibrational frequencies of the ReSe_2_ unit cell at the Γ point. d,e) Reproduced with permission.^97^ Copyright 2014, the authors, published under CC‐BY‐4.0 license. f) Optical absorption spectra of monolayer ReS_2_ and ReSe_2_ for the incident light polarized along the Γ‐K_2_ direction and the Γ‐M_3_ direction. g) Polarization‐dependent oscillator strength of excitons in monolayer ReS_2_, the exciton E_Γ_, the exciton E_Λ_ and their combined oscillator strength in monolayer ReSe_2_ with a 50 meV smearing to mimic the absorption spectrum. Reproduced with permission.^65^ Copyright 2015, American Physical Society.

Raman spectroscopy is a useful optical method for investigating lattice vibrations and elementary excitations and has been extensively used for the characterization of anisotropic 2D materials.^13^, ^97^ In TMDs, Raman spectroscopy can be applied to verify the crystal phases and distinguish the layer numbers.^97^ In addition, the Raman response of in‐plane anisotropic TMDs is related to the relative relations between the crystalline orientation and the polarizations of the incident laser and Raman scattered photons.^104^ In this section, we discuss the Raman spectra of 1T′ (or Td)‐phase TMDs, which show the anisotropy of the TMDs. Taking MoTe_2_ as an example, the Raman spectra of the 2H phase (black) and the 1T′ phase (red) of MoTe_2_ are shown in **Figure** **3**a. 2H MoTe_2_ exhibits E_2g_ and A_g_ modes at 235 and 174 cm^−1^, while 1T′ MoTe_2_ does not exhibit an E_2g_ mode, but instead new peaks appear at 124, 138, and 272 cm^−1^.^105^, ^106^ The appearance of these new peaks indicates that the 1T′ phase has relatively lower in‐plane symmetry than the 2H phase. The lower symmetry is fundamental in the anisotropic properties of MoTe_2_. The Raman spectra of mono‐ to few‐layer TMDs in the 1T′ (or Td) phase (MTe_2_, and ReX_2_) are shown in Figure 3b–d.

Moreover, for ReX_2_, Raman scattering of the linearly polarized exciton also verifies the strong anisotropy. Chenet et al. measured the Raman spectrum of ReS_2_ in a backscattering geometry using a laser of 532 nm with a fixed linear polarizer. The strongest modes are present between 120 and 240 cm^−1^ in unpolarized Raman spectrum for a ReS_2_ monolayer.^57^ Experimentally, Wolverson et al. found that the ReSe_2_ Raman modes occupy the frequency range from 100 to 300 cm^−1^ and are densely spaced, with the exception of a gap at ≈140 cm^−1^ (Figure 3e).^97^

In addition, the absorption spectrum further confirms the strong anisotropy of the excitonic transition. The absorption spectrum is anisotropic with respect to the polarization direction of the incident light.^107^ For monolayer ReS_2_, its low symmetry caused an anisotropic optical response that can be described as the single‐particle optical absorption level. As shown in Figure 3f, for the incident light polarized along the Γ‐K_2_ (*x*) direction, the single‐particle optical absorption spectrum begins near 2.7 eV, while for the Γ‐M_3_ (*y*) direction, the spectrum becomes significant at 2.9 eV. Ho and Huang reveal that this optical anisotropy is assigned to the transitions from nonbonding Re 5d t_2g_ to 5d t_2g_* and to antibonding chalcogen p and σ states.^107^ Monolayer ReSe_2_ exhibits a similar anisotropic optical response, with subtle differences.^65^ The optical absorption spectrum direction of monolayer ReSe_2_ is different from the polarization direction of incident light. The lowest‐energy absorption peak (≈1.4 eV) along the Γ‐K_2_ (*x*) direction does not completely disappear for the incident light polarized along the Γ‐M_3_ (*y*) direction. Zhong et al. verified that the lowest‐energy peak of ReSe_2_ consists of two excitons (E_Γ_ and E_Λ_), in contrast to that of ReS_2_. E_Γ_ is dark when the incident light is polarized along the Γ‐M_3_ (*y*) direction, such as the lowest exciton in ReS_2_. Differently, E_Λ_ is not completely dark along the same direction. Thus, the lowest‐energy peak does not have an extremely high polarization anisotropy. The oscillator strength of the excitons (E_Γ_, E_Λ_) shows significant spatial anisotropy. However, the prominent absorption peak at 1.4 eV is the combination of these two excitons, and the overall optical absorption does not show complete anisotropy (Figure 3g).^65^

## 3 Preparation Methods

Although the 2D TMDs MTe_2_ and ReX_2_ have the abovementioned anisotropic structures, the large‐area integration of these materials in the numerous methods that produce single‐crystal and uniform‐thickness films is limited. Most devices involving isotropic photoresponses have <5 nm layer thickness, that is, the controlled large‐area synthesis of few layered TMDs is a prerequisite for utilizing these anisotropic properties. Up to now, many methods have been designed to obtain high‐quality anisotropic TMDs. In general, the preparation methods, such as mechanical exfoliation^109^ and chemical vapor deposition,^110^, ^111^ can be divided into top‐down and bottom‐up methods. In this section, we give a brief review of the mechanical exfoliation and CVD methods to obtain MTe_2_ and ReX_2_ nanosheets.

### 3.1 Mechanical Exfoliation

The mechanical exfoliation method (or Scotch‐tape method) was first discovered by Novoselov et al., who used the method to obtain 2D graphene.^112^ This method was a watershed in 2D material research, which may take the center stage in tomorrow's technology. Despite its simplicity and crude procedure, the as‐cleaved materials provide crystalline samples with extraordinary mechanical and electrical properties.^112^, ^113^, ^114^ Similar to graphene, anisotropic materials of MTe_2_, and ReX_2_, can also be mechanically exfoliated from a natural crystalline sample to study the fundamental properties of structural anisotropy, and findings suggest that these monolayers may offer unique applications in polarized photodetectors, sensors, and photonic devices, which has attracted a wide range of attention.^28^, ^40^, ^115^, ^116^, ^117^, ^118^, ^119^ For instance, Octon et al. obtained fast, high‐responsivity, few‐layer MoTe_2_ photodetectors by mechanical exfoliation.^22^ Liu et al. prepared high‐responsivity phototransistors based on few‐layer ReS_2_ for weak signal detection by a standard mechanical exfoliation method.^83^ Lu et al. obtained high‐performance ReS_2_ nanosheet photodetectors via mechanical exfoliation from bulk Mo:ReS_2_, which showed different performances in different gas environments.^120^ In particular, to further understand the processes of exfoliation, Golberg et al. systematically investigated the cleavage processes and associated mechanical behaviors via a direct in situ transmission electron microscopy (TEM) probing technique.^121^ The results showed that the bending behavior of atomic layers is related to the number of layers during exfoliation.^121^, ^122^ For bulk layered materials, one common deformation mechanism is the formation of kinks.^123^ However, when the atomic system has <11 layers, the equilibrium shape is determined by noncovalent dispersion forces that constitute the surface energy. Undoubtedly, mechanical exfoliation method has allowed for substantial progress in elucidating the basic properties and applications of TMDs. However, mechanical exfoliation is likely to produce edges and ribbons along well‐defined crystalline directions,^124^ and the layers, morphology, and edges are still not controllable. Additionally, to realize large‐area fabrication, the mechanical exfoliation method seems to face significant challenges because of the extremely low yield and low controllability of the layer number and large‐area uniformity. Therefore, this technology is expected to have limited commercial high‐end applications and to only be used for scientific research.^125^

### 3.2 CVD

CVD is a well‐established technology that has been demonstrated as a facile method for synthesizing large‐scale monolayer crystals, including graphene, MX_2_,^73^, ^110^, ^126^, ^127^, ^128^, ^129^, ^130^, ^131^, ^132^, ^133^, ^134^ and their heterojunctions.^135^, ^136^, ^137^, ^138^, ^139^, ^140^ Compared with the exfoliation method, the direct synthesis of few‐layer and monolayer MX_2_ by CVD is critical to large‐scale applications. In this section, we discuss the synthesis of anisotropic materials of MTe_2_, and ReX_2_, by CVD methods.

**Figure 4:**
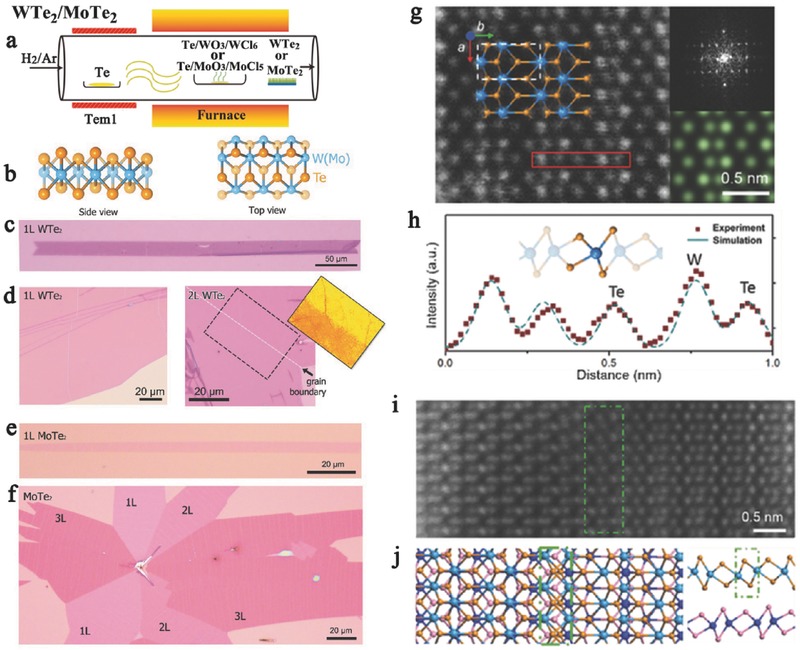
a) Illustration of WTe_2_ /MoTe_2_ growth modes via CVD system. b) Side and top views of the crystal structure of 1T′ W(Mo)Te_2_, respectively. c) Optical image of a large single crystalline WTe_2_ monolayer. d) WTe_2_ monolayer film and an optical image of a large bilayer WTe_2_ with grain boundary, highlighting the location of the grain boundary. e) Optical image of a single crystalline MoTe_2_ monolayer, with the length and width of 150 and 8 µm, respectively. f) Optical image of a MoTe_2_ flake containing 1L, 2L, and 3L MoTe_2_. The number of layer can be easily identified by their contrast. g) Scanning transmission electron microscopy (STEM) Z‐contrast image of a monolayer WTe_2_. The coordinate and structural model are overlaid on the image. Insets: Fast Fourier‐transform (FFT) pattern and simulated STEM image of the monolayer WTe_2_. h) Line intensity profile file of the region highlighted by red rectangle in panel (g). i) STEM Z‐contrast image of an atomically sharp stacking boundary between the 2H (left) and 2H′ (right) stacking. j) The structural model is optimized by DFT calculations. Reproduced with permission.^146^

There are two main obstacles for the synthesis of WTe_2_ and MoTe_2_ by existing CVD or physical vapor deposition methods. First, the electronegativity difference between the transitional metal (W or Mo) and Te (0.4 or 0.3 eV) is low.^141^ The poor electronegativity difference between the transitional metal (W or Mo) and Te indicates the existence of weak bonding between the metals and Te atoms, which makes the WTe_2_ or MoTe_2_ stoichiometry difficult to obtain. Another problem involves synthetic issues of both the precursors and products, including oxidation,^141^ volatility,^142^ thermal instability,^143^ and phase targeting. In general, W(Mo)Te_2_ single crystals are grown by the chemical vapor transport (CVT) method, and then, few‐layer flakes are exfoliated onto SiO_2_/p++ Si substrates.^144^ Until now, few papers have focused on the synthesis of telluride, particularly the synthesis of single‐crystal monolayers by CVD. In 2015, Park et al. and Zhou et al. synthesized polycrystalline MoTe_2_ thick films via the tellurization of Mo films deposited on 300 nm thick SiO_2_/Si substrates with an e‐beam evaporator or sputterer.^63^, ^145^ However, the quality of this as‐grown MoTe_2_ is inferior to that of the mechanically exfoliated sample. This method is difficult to apply to the synthesis of WTe_2_ due to the low chemical reactivity between W and Te. Gong et al.'s work indicated that Te can facilitate the synthesis of MoS_2_/WS_2_ heterojunctions by lowering the melting point of the materials.^136^ Inspired by this phenomenon, Zhou et al. demonstrated an effective CVD strategy, as shown in **Figure** **4**a,b using a Te:metal oxide weight ratio of 1:1:1, to directly synthesize few‐layer and monolayer WTe_2_ and MoTe_2_ on a large scale. As shown in Figure 4c–f, the structures of the as‐grown WTe_2_ and MoTe_2_ monolayers were characterized by their optical vibrational modes in Raman spectroscopy, and a low defect concentration was confirmed (Figure 4g–j).^146^ This effective route may lay the foundation for the construction of atomically thin telluride materials, the realization of fundamental properties, and the large‐area integration of current silicon substrates.

**Figure 5:**
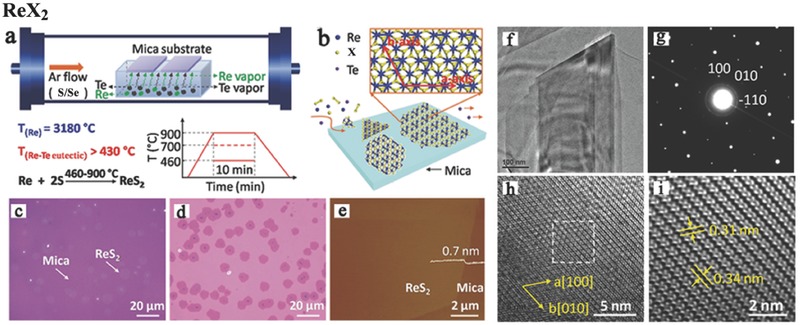
a) Illustration of ReX_2_ (X = S, Se) growth modes via CVD method. The tellurium‐assisted CVD growth approach and b) the surface reaction approaches during epitaxial growth process of ReX_2_ atomic layer on mica. Optical images of monolayer ReS_2_ grown on c) mica substrate, d) after transferred onto SiO_2_/Si (300 nm) substrate. e) Atomic force microscope (AFM) image of as‐grown ReS_2_ on mica substrate. f) Low‐resolution TEM image of the ReS_2_ supported on a TEM grid. g) Selected area electron diffraction (SAED) patterns of the ReS_2_. h) High‐resolution TEM image of the ReS_2_. i) Fast Fourier‐transform (FFT) image of the marked area in (h). Reproduced with permission.^62^ Copyright 2016.

The melting point of Re is ≈3180 °C, which is one of the highest melting point of all metals, and S and Se have relatively low melting points (155 and 221 °C, respectively) and high vapor pressures.^59^ Thus, the direct crystal growth of ReX_2_ is relatively challenging because of the large difference in melting points. Previously, ReX_2_ has been synthesized in bulk crystals via CVT^125^ or via halogen vapor transport using I_2_ or Br_2_ as a transport agent.^90^, ^119^, ^148^, ^149^, ^150^, ^151^, ^152^ It is inevitable that unintentional background doping (I_2_ or Br_2_) may change the electrical properties of the materials. The crystals grown by the I_2_ vapor transport technique are typically p‐type,^148^, ^149^ while the use of Br_2_ usually results in n‐type materials.^90^, ^119^, ^151^, ^153^ Until now, studies on the synthesis of TMDs via CVD are still rare. Very recently, many groups have explored easier and more controlled fabrication methods that give higher yields of ReS_2_ and ReSe_2_ nanosheets.^115^, ^154^, ^155^ As shown in **Figure** **5**a,b, Cui et al. introduced a tellurium material^62^ based on the Re—Te binary eutectic, whose eutectic point can be lowered to 850 °C or even to 430 °C when the Te—Re weight ratio is 90%. Those novel strategies assist the epitaxial growth of large‐area, highly crystalline ReS_2_ atomic layers on mica substrates (Figure 5c–i).^62^ Similarly, Keyshar et al. fabricated ReS_2_ at a low growth temperature (450 °C) with ammonium perrhenate and sulfur as the raw materials on SiO_2_/Si substrates.^132^ Wu et al. observed the domain architecture and grain boundaries of ReS_2_ on sapphire substrates,^154^ while Zhai et al. and co‐workers achieved the growth of hexagonal single‐crystalline ReS_2_ flake for the first time.^84^ Large‐area continuous polycrystalline bilayer ReS_2_ films were grown via CVD in a three‐zone horizontal tube furnace using ReO_3_ and S. A similar route using Se as a precursor material on SiO_2_/Si substrates was reported by Hafeez et al.^84^ Interestingly, sapphire was found to facilitate thinner flake or film growth, similar to WSe_2_ on sapphire substrates,^141^ but the cause is still unclear.

### 3.3 Other Methods

Chemical, electrochemical, and liquid‐phase exfoliation are also effective synthetic methods providing the scalable production of stacked 2D thin films and heterostructures, which have wide application in research fields such as catalysis^154^ and energy storage^156^ and in FETs.^157^ For example, Fujita et al. produced chemically exfoliated ReS_2_ nanosheets from bulk powders with a solvent‐free method by lithium intercalation. Meanwhile, their semiconducting nature and photocatalytic properties were retained.^158^ Recently, Sun et al. reported a novel low‐temperature solution synthesis of few‐layer 1T′ MoTe_2_ nanostructures by the solvothermal method. However, these nanostructures exhibit a lateral lattice compression of ≈1% compared with the bulk analogue, causing light compressive lattice strain.^159^ Molecular beam epitaxy (MBE) has the unique advantages over other growth methods of elementally controlled deposition rates and easy switching from one material to another. MBE is also used by some groups to realize the precisely controlled growth of high‐quality 2D structures or heterostructures.^160^ However, high instrument costs make the commercial viability of MBE technology difficult.

## 4 Applications for 2D Optoelectronic and Electronic Devices

Logic devices based on metal–oxide–semiconductor field‐effect transistors (MOSFETs) are fundamental in microelectronic circuits.^161^, ^162^ As the feature size decreases, the degradation of the MOSFET due to short channel effects is inevitable.^163^, ^164^ TMDs are favored for their dangling‐bond‐free morphology and atomic‐scale thickness. As a consequence, its carrier scattering is negligible, while its carrier density can be easily controlled via the gate voltage. In this section, we discuss the recent developments of FETs and photodetectors based on anisotropic TMDs. We first investigate the electronic transport phenomena and application of few‐layered MTe_2_ and ReX_2_. Then, MTe_2_ and ReX_2_ photodetectors based on photovoltaic effects in the near‐IR and visible range for application in optoelectronics are discussed. Notably, polarized light detection is demonstrated in orientation‐dependent field‐effect photodetectors. Furthermore, we discuss the different optoelectronic behaviors in vdW heterostructures with various composite structures and tuning of the photodetector through intercalation and atmospheric exposure. Finally, we propose prospects for piezoelectric and thermoelectric applications.

### 4.1 FETs Based on Anisotropic TMDs

FETs, one of the most elementary components in electronics, are widely used in a variety of electronic components. However, the traditional semiconductor FET is close to the miniaturization limit under the constant exploration of many researchers.^7^ Sub‐10 nm channel lengths lead to multiple challenges in traditional FETs, such as drain‐induced barrier lowering and punch through, surface scattering, velocity saturation, impact ionization, and hot electron effects.^163^, ^165^ The atomically flat basal planes of 2D TMDs are ideal for electronic device construction.

**Figure 6:**
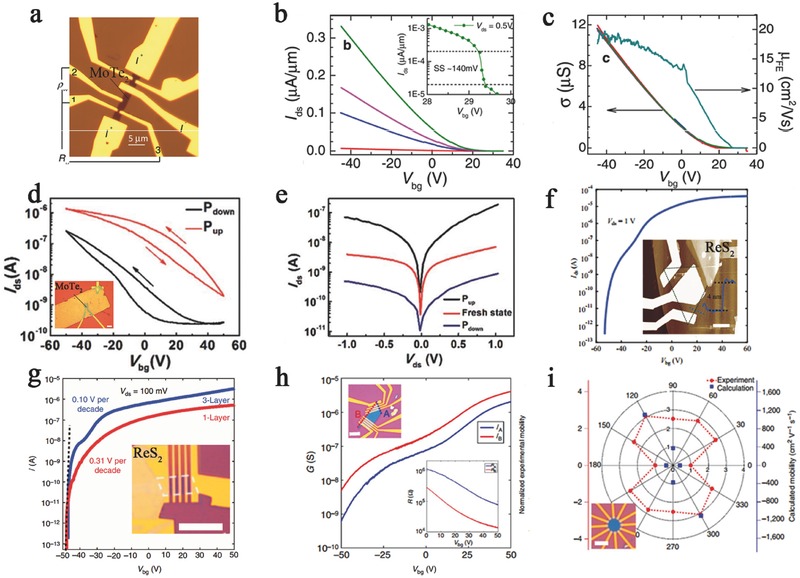
a) Optical image of bilayered MoTe_2_‐based field‐effect transistors. b) The linear curves of *I*_ds_–*V*_ds_ applied by different bias. The inset shows a subthreshold swing SS ≈140 mV, when *I*_ds_ as a function of *V*_bg_ in a limited scale. c) Conductivity (left axis) and mobility (right axis) as a function of *V*_bg_ varied from 40 to −40 V. Reproduced with permission.^167^ Copyright 2014, American Chemical Society. d) The back‐gate transfer curves of MoT_e2_‐based phototransistors under the P_up_ state (red curve) and P_down_ state (dark curve) of top gate fixing *V*_ds_ at 1 V. Inset: Image of few‐layer MoTe_2_‐based FET with a ferroelectric polymer top gate. e) The output characteristics with fresh state (ferroelectric layer without polarization), P_up_ state, and P_down_ state. Reproduced with permission.^75^ Copyright 2016, Royal Society of Chemistry. f) The transfer curve of a ReS_2_ transistor at a fixed *V*_ds_ value of 1.0 V. Inset: AFM image of ReS_2_ device. Reproduced with permission.^83^ g) Transfer curves of monolayer (red) and trilayer (blue) ReS_2_ FET devices, when *V*_ds_ is fixed at 100 mV. h) Transfer curves of anisotropic ReS_2_ FETs along A and B direction of a five‐layer flake (with an inner angle of 60° or 120°. Top inset shows optical image of the devices and the inset in the bottom performs the four‐probe resistance under *V*_bg_ varying between 0 and 60 V. i) The calculated (red dots) and experimental (blue dots) mobility of monolayer ReS_2_ is plotted in the same graph, and the lowest mobility direction was set to be the 0° (or 180°) reference. Inset: The optical image of the device. Reproduced with permission.^67^ Copyright 2015, the authors, published under CC‐BY‐4.0 license.

Most MTe_2_‐based FETs have been reported to possess a carrier mobility of 1–68 cm^2^ V^−1^ s^−1^ and large on/off ratios of 10^4^–10^6^.^16^, ^22^, ^75^, ^98^, ^166^, ^167^, ^168^ Pradhan et al. reported hole‐doped MoTe_2_ field‐effect transistors (**Figure** **6**a) with a saturated carrier mobility of up to 20 cm^2^ V^−1^ s^−1^ under a suitable bias at room temperature. This device displayed an on/off ratio over 10^6^ and typical subthreshold swings of ≈140 mV dec^−1^ (Figure 6b,c).^167^ Specifically, a few‐layer MoTe_2_‐based phototransistor tuning by a ferroelectric polymer poly (vinylidene fluoride‐trifluoroethylene‐chlorofluoroethylene) (P(VDF‐TrFE) top gate reached a current maximum carrier mobility of 68 cm^2^ V^−1^ s^−1^ among MoTe_2_‐based FET (Figure 6d). This ultrahigh carrier mobility shows that intercalation tuning can efficiently enhance the field‐effect transistor performance. When the top gate voltage was swept from −35 to 35 V at room temperature, a high on/off ratio of 10^5^ with a fixed source–drain voltage of 1 V was observed (Figure 6e).^75^ Similar to MTe_2_, the high carrier mobility and on/off ratio of ReX_2_‐based FETs was reported to reach 5–40 cm^2^ V^−1^ s^−1^ and 10^4^–10^8^, respectively (Figure 6f).^28^, ^33^ The high‐performance ReS_2_ devices showed excellent n‐type FET behavior with a high electron carrier mobility of 30 cm^2^ V^−1^ s^−1^ and an off state current smaller than 1 pA. And the on/off ratio of 10^8^ is comparable with the highest level achieved with MoS_2_. In addition, angle‐resolved FET has been implemented by regulating asymmetrical monolayer ReS_2_. Liu et al. observed that the anisotropic ReS_2_‐based FET under a fixed source–drain bias voltage of 100 mV had a large on/off ratio of 10^7^ and low subthreshold swings of 100 mV dec^−1^ (Figure 6g), when the back gate was swept from −50 to 50 V. The anisotropic mobility ratio µm_ax_/µm_in_ along two principle axes of this device is 3.1, which is better than that of a device containing few‐layer black phosphorus (Figure 6h).^27^, ^41^ The direction of lowest mobility (0° or 180°) was defined as the reference direction, and the highest mobility of 15.4 cm^2^ V^−1^ s^−1^ was measured in a six‐layer device in the 120° (or 300°) direction (Figure 6i).^67^

### 4.2 Development of 2D Anisotropic Photodetectors

Light detection is one of the basic components of many optoelectronic applications, including imaging, quantum communication, dynamic capture technology, positioning, and guidance.^4^, ^169^, ^170^, ^171^ Modern photodetector devices require small sizes, fast response times, and high detection accuracy and sensitivity over a wide wavelength range. 2D materials are ideal building blocks for photodetectors because of their plasticity, controllability, and excellent physical properties.^172^, ^173^ In the application of 2D TMDs in photodetectors, there are two main photon–matter interaction operating modes: the photoconduction mode and the photocurrent mode, based on the photovoltaic effect.^12^ In the photoconduction mode, photoexcited carriers directly increase the device's conductance. But in the photocurrent mode, photoexcited carriers are transformed into current under an asymmetric built‐in electric field.^12^ A high responsivity of 880 A W^−1^ at a wavelength of 561 nm based on the photoconduction effect was found in monolayer MoS_2_.^23^ It has been reported that the photocurrent in vertically stacked graphene/MoS_2_ (16 nm)/graphene heterostructures can be modulated by the gate bias to achieve high quantum efficiency (MAX_EQE_ = 55%, MAX_IQE_ = 85%).^174^ Although TMDs such as MoS_2_, MoSe_2_, WS_2_, and WSe_2_ have led to considerable progress in the field of photodetectors, the optoelectronic applications of the TMD family require further in‐depth exploration. Compared to the isotropic behavior of the well‐studied TMDs, anisotropic properties can be introduced by reducing the lattice symmetry. For instance, the electronic and phonon properties of strongly anisotropic MTe_2_ and ReX_2_ are dependent on the in‐plane orientation.^41^, ^175^ These different properties along different crystal orientations are enhanced when transistors are built along a specific direction. In the photoconduction operating mode, anisotropic MTe_2_ and ReX_2_ can control the crystal orientation to enhance the separation of photogenerated electron–hole pairs.^8^, ^22^, ^76^, ^115^ This characteristic can increase the photoconductive sensitivity and achieve an ultrahigh responsivity and external quantum efficiency (EQE) under precise regulation. In the photocurrent operating mode, the regulated crystal orientations of anisotropic materials can tune the photogenerated current very well by changing the built‐in electric field at junctions to both improve the responsivity and control the wavelength range of light detection.^16^, ^87^, ^166^ We provide the performance of various photodetectors in **Table** **2** and discuss them further in the following sections.

**Table 2:** Performance parameters of photodetector (ML: Multilayer, IR: Infrared)

Devices	Responsivity [A W^−1^]	EQE [%]	Rise time [ms]	Decay time [ms]	Wavelength	References
MoTe_2_(4L)	6	–	0.16	0.3	Visible/IR	123
MoTe_2_(ML)	2560	–	–	–	Visible	76
MoTe_2_(ML)	0.024–0.05	–	1.6	1.3	Visible/IR	176
ReS_2_(ML)	16.14	3168	≈5 × 10^4^	≈5 × 10^4^	Visible	62
ReS_2_(ML)	1000	–	–	–	Visible	28
ReS_2_(ML)	88 600	2 × 10^7^	–	–	Visible	97
ReS_2_(ML)	604	1.5 × 10^5^	2	2	Visible	84
ReS_2_(monolayer)	12	–	–	–	Visible	85
ReS_2_(ML)	10^7^	–	670	5600	Visible	177
ReSe_2_(ML)	95	18 645	68	34	Visible	88
Mo:ReSe_2_	55.5	10 893	96	340	Visible	120
α‐MoTe_2_/MoS_2_	0.037–0.322	85	25	25	Visible/IR	16
P(VDF‐TrFE)/MoTe_2_	0.0164	–	1.4	1.3	IR	75
MoTe_2_/graphene	0.02	–	30	30	Visible	178
ReSe_2_/MoS_2_	6.75	1266	80	80	Visible	87

#### 4.2.1 Photodetectors Based on 2D MTe_2_ and ReX_2_

**Figure 7:**
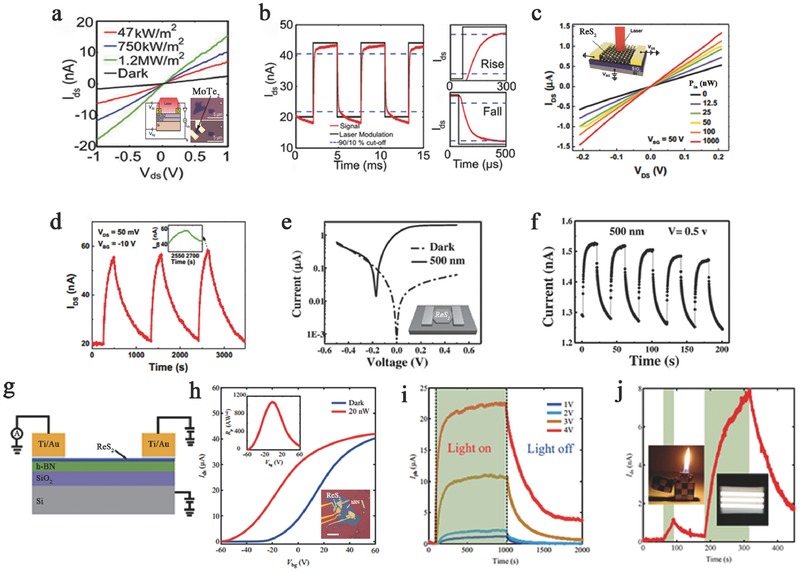
a) The curves of *V*_ds_–*I*_ds_ fixed at *V*_g_ = 0 V under a different power of 685 nm laser. Inset: The schematic and two optical images of MoTe_2_ few‐layer FET. b) Photoresponse occurred in MoTe_2_ device at *V*_ds_ = 5 V, *V*_g_ = 0 V under a laser of 178 Hz and 20 µW. Zoomed region shows a rise time and a fall time. Reproduced with permission.^22^ c) Output characteristics of the curve of the ReS_2_ back‐gate device under different incident laser powers. Inset: The schematic structure of ReS_2_ photodetector. d) Photoresponse occurred with time‐dependent *I*_ds_ of the device under an intermittent laser illumination. Inset: Enlarged image of the photoresponse peak over a small range. Reproduced with permission.^115^ e) The characteristic *I*–*V* curves were illuminated by light of the single ReS_2_ device. Inset: The schematic image of the device. f) Photoresponse occurred in ReS_2_ polycrystalline bilayer film with photocurrent under 500 nm incident light. Reproduced with permission.^84^ g) The schematic of the few‐layer ReS_2_ phototransistors. h) Linear transfer curves in the dark state and under illumination. Inset: Photoresponsivity as a function of *V*_bg_ at the top and optical microscopy image and AFM image of the same device at the bottom. i) Photoswitching behaviors with high photoresponsivity under various *V*_ds_. j) Weak signal detection in a five‐layer ReS_2_ phototransistor, fixing *V*_bg_ at −50 V and *V*_ds_ at 2.0 V. Reproduced with permission.^83^

MTe_2_ is a group VI TMD with a layer‐dependent bandgap, meaning that it has a tunable range of detection. In addition, Zhang et al. predicted that MoTe_2_ possesses a theoretical ultrahigh mobility of up to 2526 cm^2^ V^−1^ s^−1^, which is several times higher than that of MoS_2_.^8^ A high mobility indicates that the absorbed photons more quickly convert into electrical signals, which can improve the responsivity and reduce the photoresponse time. Octon et al. observed a fast photoresponse time (≈160 µs) in few‐layered MoTe_2_ (6.5 nm) photodetectors based on photoconduction under 685 nm laser illumination with a high responsivity of 6 A W^−1^ (**Figure** **7**a,b).^22^ However, the mobility is restricted due to the high contact resistance between the electrode and MoTe_2_. Yin et al. investigated the contact resistance of different electrode materials.^76^ This work revealed the emergence of a Schottky barrier in Au‐contacted devices. Strikingly enhanced electron injection from the electrode to the channel was observed due to the introduction of a tunneling mechanism. The highest electron mobility of this FET approached ≈25.2 cm^2^ V^−1^ s^−1^, with a high photoresponsivity of 2560 A W^−1^ under the illumination of a 473 nm laser.

In contrast to group VI TMDs, few‐layer ReX_2_ is a direct bandgap semiconductor with weak interlayer coupling. Thus, ReX_2_‐based applications do not require a single‐layer structure, reducing the cost and difficulty of preparation. In addition, multilayer ReX_2_ can be used in device construction to improve the light absorbance. Because of this distinct bandgap feature, ReS_2_‐based photodetectors generally have high responsivity (>10 A W^−1^) and EQE (>1000%) and thus can be applied to detect extremely weak signals. Zhang et al. first fabricated few‐layer ReS_2_‐based back‐gate photodetectors (Figure 7c,d) with a maximum gate‐tunable responsivity of 16.14 A W^−1^ and an EQE of 3168%.^115^ The photodetectors were irradiated by a focused laser beam (633 nm, 12.5–1000 nW) under 50 V back‐gate bias. In addition, the transport properties were studied at different temperatures using four‐terminal back‐gated devices, and a maximum mobility of ≈8 cm^2^ V^−1^ s^−1^ was acquired at 120 K. Although the responsivity of this device is comparable to that of graphene and MoS_2_, its applications are limited due to the slow response time (≈500 s) and small on/off ratio of 2.8. In 2016, a fast‐response‐time photodetector based on ReS_2_ flake was constructed by Hafeez et al. In their study, CVD‐grown ReS_2_ film‐ and ReS_2_ flake‐based photodetectors were illuminated at a 500 nm light of 3.11 mW cm^−2^ (Figure 7e,f). Both the rise and decay times of the ReS_2_ film‐based devices are much slower than that of the flake‐based devices. The flake‐based devices show a fast response time of 2 ms, high responsivity of 604 A W^−1^, and EQE of 1.50 × 10^5^%.^84^ In addition, Liu et al. demonstrated an ultrahigh photoresponsivity of 88600 A W^−1^, corresponding to a high EQE of 2 × 10^7^% at a wavelength of 532 nm in few‐layer ReS_2_ phototransistors (Figure 7g,h).^83^ Such high responsivity is a record for individual 2D photodetectors and is two orders of magnitude higher than that of monolayer MoS_2_,which can be used for weak signal detection (Figure 7i,j). This high photoresponsivity is attributed to an increase in photon absorption and a photogain mechanism involving trap states in ReS_2_, where the trap state density was estimated to be 1.96 × 10^13^ cm^2^ by studying the temperature‐dependent field‐effect mobility.

#### 4.2.2 Polarized Light Photodetector

**Figure 8:**
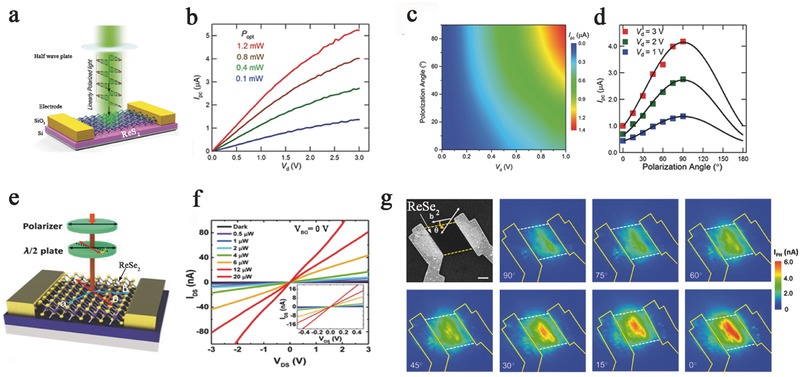
a) The schematic of the ReS_2_‐based photodetection device under illuminating polarized light by the half wave plate. The photocurrent response as a function of drain voltage under b) different light intensity green light illumination, c) different polarization light illuminations, and d) different drain biases. Reproduced with permission.^28^ e) The schematic structure of ReSe_2_ photodetectors. The half‐wave plate was used to change the polarization direction. f) *I*_ds_–*V*_ds_ of the photodetector with incident laser power varied from 0 to 20 µW. Inset: Small range of *I*_ds_–*V*_ds_ with different incident laser powers. g) Scanning electron microscope (SEM) image of the polarization‐dependent photocurrent mapping of ReSe_2_ photodetector, showing prominent linear dichroic photodetection. Reproduced with permission.^53^ Copyright 2016, American Chemical Society.

Polarized light detectors are essential components for controlling the vibration direction and period of light.^179^ This photodetector resolves the polarization direction and has enormous potential in communication, remote sensing, and photography.^180^ Therefore, polarized light photodetectors with high sensitivity and integration level are necessary. However, current devices are still far from satisfying commercial requirements. Emerging 2D materials have made progress in the detection of polarized light because of their orientation‐dependent crystal structures. Liu et al. developed a new method to detect polarized light using 2D anisotropic ReS_2_ (**Figure** **8**a). This transistor shows an ultrahigh responsivity of 10^3^ A W^−1^ in a linear dichroic photodetector with a high electron mobility of 40 cm^2^ V^−1^ s^−1^ and on/off ratio of over 10^5^ (Figure 8b–d). This study demonstrated that anisotropic ReS_2_ can be applied for light polarization detection with excellent performance, which may open new avenues for polarized light detection.^28^ Similar results have also been found in ReSe_2_ FETs, where an ambipolar gate‐tunable linear dichroism photodetector was exhibited in Figure 8e. High on/off current ratio of up to 10^7^ with stable saturation current was shown in this device (Figure 8f,g). Moreover, the mobility can be tuned by the temperature, and over 500‐fold and 100‐fold enhancements in the electron and hole mobilities, respectively, were observed at low temperatures.^53^

#### 4.2.3 Photodetector Based on Heterostructures

A photodetector based on the photocurrent operating mode consists of a tunable junction, and the current study of MTe_2_ and ReX_2_ focuses on out‐of‐plane heterostructures.^5^, ^12^, ^166^, ^181^, ^182^, ^183^ Heterostructures play a major role in conventional semiconductor technologies because interfaces provide an effective and easy way to control the carrier type, density, and mobility. Mismatches in the lattice and thermal coefficients should be avoided to ensure a satisfying performance of the heterostructures.^184^, ^185^ 2D heterostructures can overcome the two aforementioned obstacles resulting from their vertically weak van der Waals bonding characteristics.

**Figure 9:**
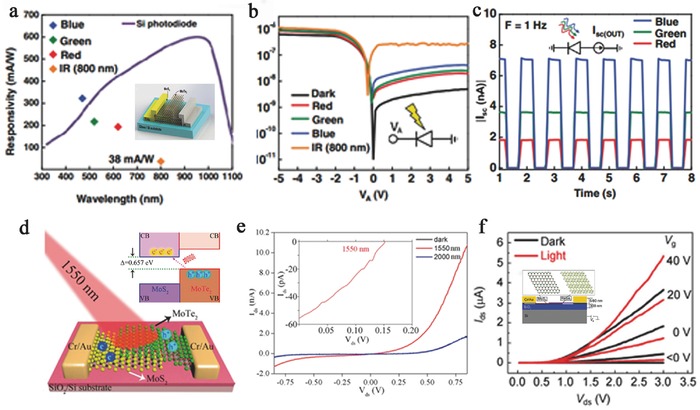
a) Photoinduced *I*–*V* curves of MoTe_2_/MoS_2_ heterojunction on a logarithmic scale along. Inset: The schematic and of MoTe_2_/MoS_2_ photodetector. b) Time domain of short‐circuit current (ISC) and under 1‐Hz RBG LED illuminations. c) Photoresponse characteristics of responsivity based on MoTe_2_/MoS_2_ photodetector under RGB LEDs and IR (800 nm) laser, obtained at zero volt. Reproduced with permission.^16^ d) The schematic illustrations of type‐II interband excitation processes in MoTe_2_/MoS_2_ vdW heterostructures. Inset: The schematic diagram of a MoTe_2_/MoS_2_ vdW heterostructure device under infrared light excitation. e) The curves of *I*_ds_–*V*_ds_ under infrared light illumination of 1550 and 2000 nm. Inset: Photovoltaic effect of the fabricated device under 1550 nm light illumination. Characterization of ReSe_2_/MoS_2_ heterojunction and device. Reproduced with permission.^166^ Copyright 2016, American Chemical Society. f) Output plots of the devices in the dark and under 8.15 mW cm^−2^ light illumination. Inset: Schematic of the device based on the p–n ReSe_2_/MoS_2_ heterojunction. Reproduced with permission.^87^ Copyright 2015, Springer.

Type‐II heterostructures can effectively tune the interlayer gap due to their staggered band alignments.^186^, ^187^ Pezeshki et al. investigated photodetectors based on α‐MoTe_2_ (3l)/MoS_2_ (3l) p–n diodes working in photocurrent operating mode (**Figure** **9**a). The calculated type‐II interlayer gap between the conduction band minimum of MoS_2_ and the valence band maximum of α‐MoTe_2_ is ≈0.35 eV. This device showed a photoresponse time within 25 ms and maintained a stable photovoltaic effect with 1–3 Hz photoswitching dynamics and a good on/off current ratio of ≈1 × 10^3^–4 × 10^3^ (Figure 9b,c). Blue to IR (470–800 nm) light was employed to examine this device, and blue photons gave the highest responsivity of 322 mA W^−1^ with 85% EQE. The responsivity and EQE of this device decreased with reduced photon energy, where the lowest values were 37 mA W^−1^ and 6% EQE under 800 nm IR illumination (Figure 9a).^16^ Furthermore, Zhang et al. studied IR photodetection in a type‐II MoTe_2_/MoS_2_ heterostructure with strong interlayer coupling by interlayer optical transition (Figure 9d). This device had an interlayer gap of 0.66 eV (≈1880 nm) and effectively detected infrared light (1.55 µm) with a distinct photocurrent response (Figure 9e).^166^ A multilayer ReSe_2_/MoS_2_ p–n heterostructure‐based photodetector operated in photocurrent mode was examined by Wang et al.'s group (Figure 9f). This mobility of the heterostructure device was obviously enhanced (electron, 4 cm^2^ V^−1^ s^−1^) and was higher than that of ReSe_2_ (hole, 0.145 cm^2^ V^−1^ s^−1^) and MoS_2_ (electron, 0.226 cm^2^ V^−1^ s^−1^). In particular, this device demonstrated an extremely high responsivity of 6.75 A W^−1^ and EQE of 1266%, which are better than other X/MoS_2_‐based photodetectors investigated so far.^87^

#### 4.2.4 Photodetector Based on Intercalation and Atmosphere Tuning

**Figure 10:**
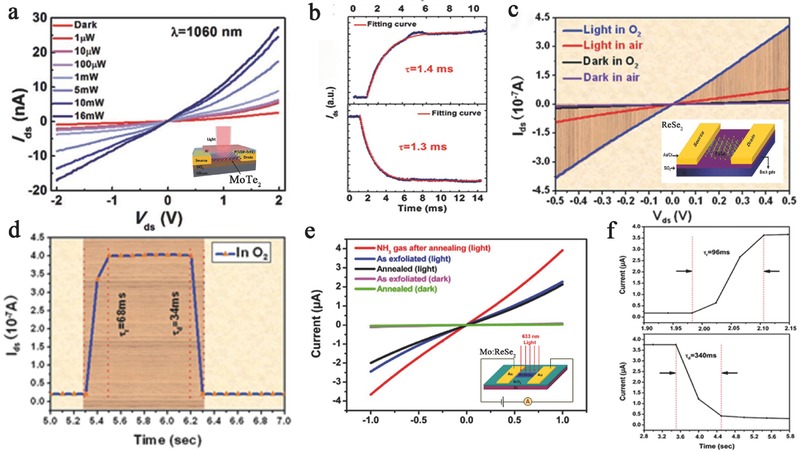
a) The output characteristics under different power of incident light. Inset: Schematic structure of P(VDF‐TrFE) top‐gate FET based on few‐layer MoTe_2_ was illuminated under a laser. b) The rise time and the fall time of photocurrent and fitting curves by exponent function. Reproduced with permission.^75^ Copyright 2016, Royal Society of Chemistry. c) The curves of *I*_ds_–*V*_ds_ measured in O_2_ or air in the dark or under red light illumination. Inset: Schematic structure of ReSe_2_‐based photodetector. d) The rise time and the fall time of photoresponse in O_2_ environment. Reproduced with permission.^88^ Copyright 2014, Royal Society of Chemistry. e) The *I*–*V* curves of the photodetector were illuminated with 633 nm light (20 mW cm^−2^) in different conditions. Inset: Schematic structure of Mo:ReSe_2_‐based FET. f) The rise time and the fall time of photoresponse under illumination of 633 nm in NH_3_ environment. Reproduced with permission.^120^ Copyright 2014, the authors, published under CC‐BY‐4.0 license.

Various photodetectors with superb performance based on MTe_2_ and ReX_2_ were discussed in the previous sections. Most photodetectors were modulated by the applied gate voltage and temperature.^85^, ^176^, ^177^, ^178^, ^188^ However, other tuning methods, such as by intercalation or atmosphere, can also enhance the physical properties of the host materials to enhance the optoelectronic performance.^14^ As mentioned above, Huang et al. fabricated a ferroelectric polymer P(VDF‐TrFE) top‐gate photodetector based on MoTe_2_, with a high carrier mobility that was calculated to be 68 cm^2^ V^−1^ s^−1^ (**Figure** **10**a,b). Because the remnant polarization of ferroelectrics reduces the dark current, this device performs over a broad photoresponse range (0.6–1.5 mm) with the maximum responsivity and detectivity reaching 16.4 mA W^−1^ and 1.94 × 10^8^ Jones, respectively, under 1060 nm light.^75^ ReSe_2_ has similar properties to ReS_2_, and ReSe_2_‐based photodetectors have extremely high responsivity and EQE. In addition, layer‐dependent electrical and optoelectronic responses have been observed in ReSe_2_‐based photodetectors. Yang et al. (Figure 10c,d) showed that a monolayer ReSe_2_‐based transistor possessed a much higher mobility (≈9.78 cm^2^ V^−1^ s^−1^) than a multilayer device and a red‐light‐sensitive (633 nm) mobility of ≈14.1 cm^2^ V^−1^ s^−1^. Furthermore, because of the enhanced molecular physisorption due to the low symmetry structure, the photoconduction of ReSe_2_ nanosheet photodetectors is significantly affected by the gas atmosphere. This atmosphere‐sensitive property allows for an additional tuning approach by gas molecule gating. A high photoresponsivity of 95 A W^−1^ and an EQE of 18645% were found in a monolayer ReSe_2_‐based transistor under red light (633 nm) and an O_2_ environment (Figure 10e,f).^88^ A similar phenomenon was also observed in Mo‐doped ReSe_2_ nanosheet photodetectors, which showed a photoresponsivity of 55.5 A W^−1^ and an EQE of 10893% in NH_3_ after annealing.^120^

### 4.3 Prospects for Piezoelectric and Thermoelectric Applications

Piezoelectricity is a direct conversion from mechanical stress force to electricity via accumulating polarization charge in asymmetrical atomic structures, which has been widely applied in micro‐electromechanical systems, actuation, sensor, and electronics calls. TMDs have been ideal candidates for piezoelectric applications for the following reasons.^189^, ^190^, ^191^, ^192^ First, their non‐centrosymmetry and low‐dimension structure is considered to induce piezoelectricity.^192^ Second, TMDs own high crystallinity which can withstand great pressure.^190^, ^193^, ^194^ Third, TMDs can retain their single‐layer piezoelectric structures without large surface energy, causing thermodynamically unstable lattice reconstruction under ambient conditions.^189^, ^194^ In 2014, the piezoelectricity of monolayer MoS_2_ was first observed with a power density of 2 mW m^−2^ and mechanical‐to‐electrical energy conversion efficiency of 5.08% by Wu et al.^190^ Then, Zhu et al.^189^ measured the piezoelectricity of monolayer MoS_2_ with a piezoelectric coefficient of 2.9 × 10^−10^ C m^−1^. Compared with MoS_2_, MTe_2_ possesses more intense asymmetry because of distorted octahedral structure.^195^ And it is important that piezoelectricity originates from non‐centrosymmetry structure.^192^ Thus, Duerloo et al.^192^ calculated that 2H MoTe_2_ has the highest piezoelectric coefficients (*e*_11_ = 2.98(clamped‐ion)/5.43(relaxed‐ion) 10^−10^ C m^−1^) and 2H WTe_2_ has the lowest piezoelectric coefficients in TMDs (*e*_11_ = 1.60(clamped‐ion)/3.40(relaxed‐ion) 10^−10^ C m^−1^). Both 2H MoTe_2_ and 2H WTe_2_ have the huge Δ*e*_11_ bigger than that of MoS_2_. In addition, the piezoelectric effect of 2H MTe_2_ may be much more abundant than the theoretical calculation due to its surprising magnitude.^192^

The anisotropic crystal structure may benefit not only the piezoelectric properties as mentioned above, but also the thermoelectric properties. Analogous to piezoelectricity, thermoelectric devices convert heat into electrical energy by the Seebeck effect.^1^ The low‐dimension materials such as nanowires, superlattices, or quantum dots have achieved excellent thermoelectric figure of merit (*ZT*) via reducing dimensionalities.^196^, ^197^, ^198^ And bulk SnSe is expected to enhance the thermoelectric efficiency due to anisotropic bonds.^199^ Thus, low‐dimension and anisotropic structure was considered to increase *ZT* and thermoelectric efficiency. The strong anisotropic TMDs of MTe_2_ and ReX_2_ meet the above requirements and have the potential to exceed anisotropic black phosphorus. Ma et al.^200^ have demonstrated strong anisotropic thermal conductivity of monolayer WTe_2_ by first‐principle calculations. WTe_2_ exhibits thermal conductivity of 9 and 20 W m^−1^ K^−1^ along two principal lattice directions in room temperature, which is higher than thermal conductivity of black phosphorus (<10 W m^−1^ K^−1^).^201^, ^202^ In addition, there may be two types of transport in TMDs including in‐plane transport in the van der Waals interface and out‐plane transport across van der Waals by a tunneling effect. ReX_2_ possesses weak interlayer coupling, which is different from strong interlayer coupling. The ratio between two transports of ReX_2_ which may result in an improvement of the *ZT*.^43^

## 5 Summary and Outlook

Herein, we reviewed the latest knowledge of the crystalline structure, preparation methods, and electronic and optoelectronic applications of novel and strongly anisotropic MTe_2_ and ReX_2_ materials. The anisotropy, caused by the distorted octahedral phase, was analyzed using various characterization methods. Mechanical exfoliation from bulk materials and CVD are the primary methods to prepare strongly anisotropic TMDs. Other methods, such as chemical and liquid‐phase exfoliation, were further discussed. However, the growth of high‐quality, large‐scale, and uniformly oriented MTe_2_ and ReX_2_ remains a challenge. In addition, the stacking of different 2D materials requires more in‐depth study to upgrade the device performance. Photodetectors containing MTe_2_ and ReX_2_ based on the photovoltaic effect show better performance than other TMDs in terms of the responsivity and EQE, especially in the detection of polarized light. Intercalation and atmosphere tuning were also demonstrated to improve the device performance. However, this improvement is not sufficient. Linear polarized excitons and selectively tunable optical Stark effects have been theoretical predicted in thin ReS_2_. ReX_2_‐based or MTe_2_‐based photodetectors and light‐emitting diodes are extremely promising devices that still require in‐depth examination. It is worth mentioning that magnetic‐field‐induced valley Zeeman splitting and polarization have been observed in monolayer MoTe_2_.^30^ Novel valleytronics made of MTe_2_ have great potential for next‐generation optoelectronic devices. Overall, the recent findings concerning anisotropic TMDs indicate broad promise in electronic and optoelectronic applications.

## Conflict of Interest

The authors declare no conflict of interest.
